# Supplementary material for: Differential contribution of steady‐state RNA and active transcription in chromatin organization
Source: EMBO Rep. 2019 Aug 26;20(10):e48068. doi: 10.15252/embr.201948068 (PMC6776903; doi:10.15252/embr.201948068)
Supplement: Supplementary file 1 — Expanded View Figures PDF [file EMBR-20-e48068-s001.pdf]

Expanded View Figures

Figure EV1. Nuclear changes upon RNase treatment in K562 and HeLa cells.

A Immunostaining of active caspase 3 and DAPI staining in RNase-treated or ActD-treated and control K562 cells.
B Immunostaining of Fibrillarin and DAPI staining in RNase-treated or ActD-treated and control K562 cells.
C DAPI staining of HeLa cells before and after crosslinking.
D Quantification of nuclear area in K562 bXL CTRL and bXL RNase A samples. *P*-value: two-tailed Student's *t*-test. The horizontal bands in the boxplots represent the median, the error bars represent ± 1.5 × IQR values, and the outer shapes represent the density of the data points. The sample numbers are indicated below.

 © 2019 The Authors

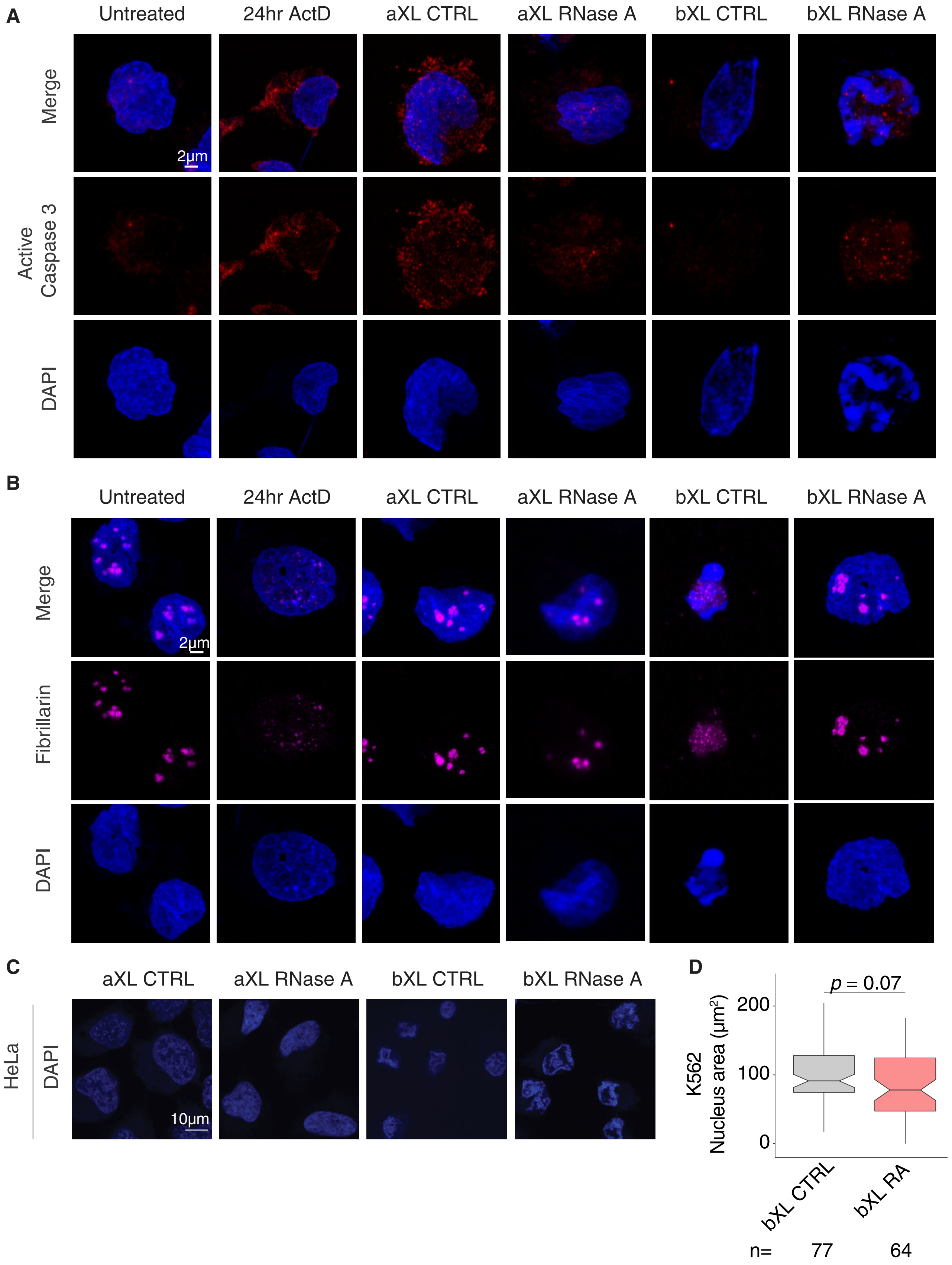
A
Untreated
24hr ActD
aXL CTRL
aXL RNase A
bXL CTRL
bXL RNase A
Merge
Active Caspase 3
DAPI
2µm
B
Untreated
24hr ActD
aXL CTRL
aXL RNase A
bXL CTRL
bXL RNase A
Merge
Fibrillarin
DAPI
2µm
C
aXL CTRL
aXL RNase A
bXL CTRL
bXL RNase A
HeLa
DAPI
10µm
D
p = 0.07
K562
Nucleus area (µm²)
200
100
0
bXL CTRL
bXL RA
n=
77
64

Figure EV1.

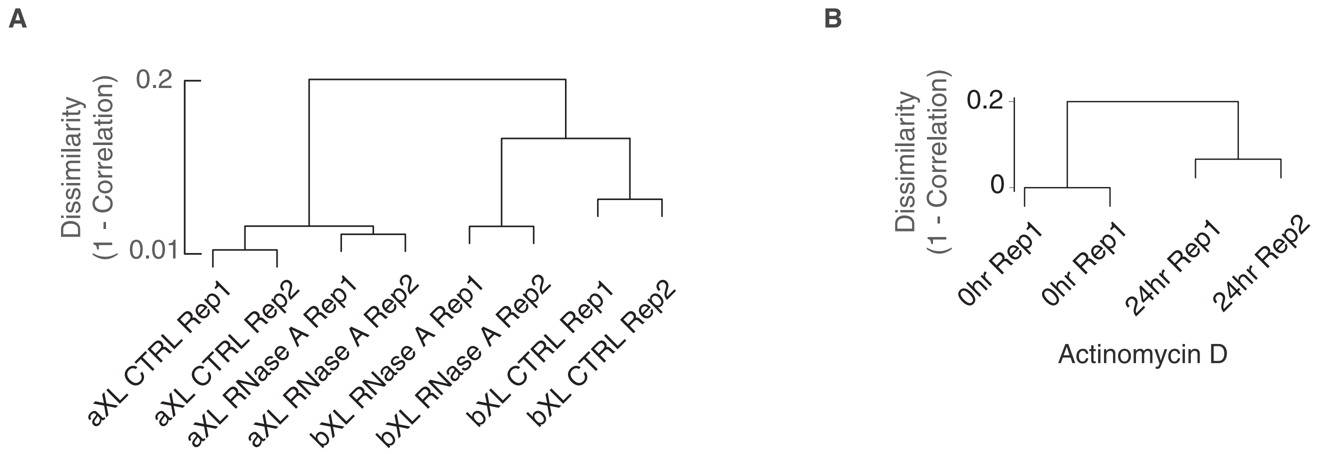

Figure EV2. Hi-C samples show high correlations.

A Dendrogram showing the Hi-C replicate dissimilarity scores (1-correlation) of RNase-treated and control samples based on 1st eigenvalues.
B Dendrogram showing the Hi-C replicate dissimilarity scores (1-correlation) of transcriptionally inhibited and control samples based on 1st eigenvalues.

 © 2019 The Authors

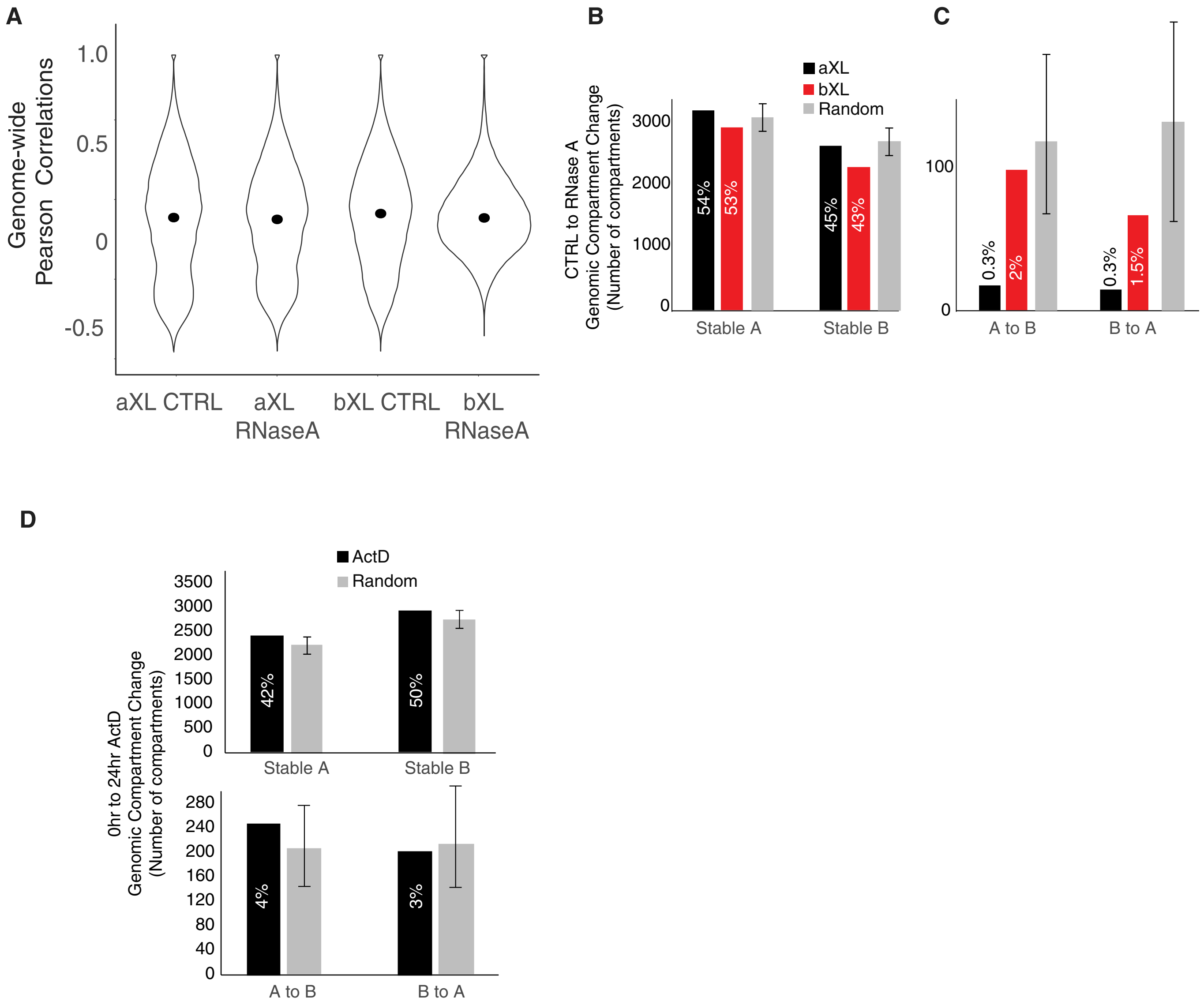

Figure EV3. Compartment switching in RNase- and ActD-treated cells.

A Violin plot showing the genome-wide Pearson correlation scores of control and RNase-treated cells, with median values depicted as black dots. The figure is prepared from the pooled Hi-C data generated with 2 biological replicates.

B, C Bar plots showing the CTRL to RNase A treatment stable (B) and switched (C) compartments for the aXL (black) and bXL (red) and randomized (gray) samples. Error bars: standard deviation.

D Bar plots showing the stable and switched compartments for control and 24-h actinomycin D-treated cells as well as the randomization averages.

© 2019 The Authors
